# Exercise duration and detraining influence not only body weight but also histopathological changes in the white adipose tissue of young male OLETF rats as an obesity model

**DOI:** 10.14814/phy2.70487

**Published:** 2025-07-26

**Authors:** Kanta Sakakura, Susumu Urakawa, Naoto Fujita

**Affiliations:** ^1^ Department of Bio‐Environmental Adaptation Sciences, Graduate School of Biomedical and Health Sciences Hiroshima University Hiroshima Japan; ^2^ Department of Musculoskeletal Functional Research and Regeneration, Graduate School of Biomedical and Health Sciences Hiroshima University Hiroshima Japan

**Keywords:** detraining, histopathological changes, long‐term exercise during young period, obesity, white adipose tissue

## Abstract

Environmental exposures during early life impact health and disease in later life. Therefore, understanding the effects of exercise during early life and detraining on obesity in adulthood may be valuable for preventing and treating obesity. This study aimed to examine the effects of short‐ and long‐term exercise and detraining during early life on the histological changes in adulthood. Four‐week‐old male Otsuka Long‐Evans Tokushima Fatty (OLETF) rats were used as an animal model of obesity. The OLETF rats were divided into the sedentary and exercise groups. The rats in the exercise group were further divided into two subgroups according to the exercise period: exercised from 4‐ to 8‐week‐old and non‐exercised from 8‐ to 20‐week‐old, and exercised from 4‐ to 12‐week‐old and non‐exercised from 12‐ to 20‐week‐old. The metabolic profiles in adulthood, such as body weight, did not significantly differ between rats subjected to short‐ and long‐term exercise during the young period. However, histological changes in white adipose tissue, such as adipocyte hypertrophy and chronic inflammation, were effectively reduced with long‐term exercise compared with short‐term exercise. Long‐term exercise during the young period resulted in low adiposity in adulthood, although no significant differences in body weight after detraining were observed.

## 1 INTRODUCTION

The prevalence of childhood obesity has increased and become a global problem in recent years. According to a 2024 survey, the global age‐standardized prevalence of obesity in school‐aged children and adolescents increased almost fourfold from 1990 to 2022, with 159 million girls and boys with obesity worldwide (NCD Risk Factor Collaboration, 2024). Childhood obesity leads to adult obesity (Singh et al., 2008). With the progression of obesity, lipids accumulate in the white adipose tissue (WAT) owing to adipocyte hypertrophy and hyperplasia, which result in chronic inflammation of the adipose tissue (Ye et al., 2022). Inflamed adipose tissue secretes inflammatory cytokines, which spread to various organs throughout the body, causing obesity‐related health problems, such as glucose intolerance and fatty liver (Kawai et al., 2021). Therefore, childhood obesity is a risk factor for the onset of obesity‐related health problems in adulthood (Juonala et al., 2011), and early intervention for childhood obesity is needed.

Binge eating and obesity are highly comorbid disorders. Binge eating is highly prevalent in children and adolescents with obesity, which is attributed to the loss of control in eating (Byrne et al., 2019). Moreover, childhood obesity is associated with several complications, such as hyperglycemia, dyslipidemia, and hepatic steatosis, which often persist into adulthood (Moreno, 2025). In children with obesity, the prevalence ratios were 1.4 for elevated fasting plasma glucose, 4.2 for high plasma triglyceride (TG), and 26.1 for hepatic steatosis compared to those in children with normal body weight (Sharma et al., 2019). Concerning long‐term consequences, persistent obesity from childhood to adulthood is associated with a higher risk of diabetes (Moreno, 2025). Therefore, therapeutic controls of not only body weight but also eating behavior during childhood are essential to prevent health complications and reduce obesity‐related health problems in adulthood.

Exercise is essential for preventing obesity. It is a subset of physical activity, defined as any body movement produced by skeletal muscle contraction, which requires energy expenditure. Regular physical exercise decreases WAT mass and ectopic fat accumulation in humans (Thyfault & Bergouignan, 2020) and rodents (Fujita et al., 2018) with obesity. However, the low physical activity level in children is a global problem (Elmesmari et al., 2018; Keane et al., 2017). The amount of time spent on regular physical activity, including exercise, decreases from childhood to adolescence (Ishii et al., 2015; Steene‐Johannessen et al., 2020). Effective management of people with obesity requires long‐term exercise, and adherence to regular exercise is essential to achieve its health benefits, such as body weight loss. Low adherence to exercise impacts treatment efficacy, which could hinder maintaining normal body weight and preventing health complications. However, low adherence to exercise for obesity is common among all generations. Adherence to the physical activity guidelines in obese adults from the United States is approximately 15% (López‐Gil et al., 2025). A systematic review has indicated that dropout prevalence of regular exercise in children and adolescents with obesity depends on the exercise duration (Guijo et al., 2024). Thus, a longer exercise period results in lower adherence. A patient with low adherence could revert back to old eating habits, and consequently, regain excess weight. Strictly, in most cases, low adherence to regular exercise during childhood results in not regaining body weight after weight reduction but inconsistent weight gain. Presently, avoiding a decrease in physical activity, including exercise, from childhood to adolescence is difficult. The influences of withdrawal from regular exercise might be different on childhood obesity and adulthood obesity; therefore, the effects of exercise duration in children should be examined with a focus on obesity and metabolism.

Several animal studies have reported the effects of exercise during the young period and discontinuation of exercise, which is detraining. Body weight gain is commonly associated with exercise cessation (Del Vecchio et al., 2020). Sertie et al. have reported that Wistar rats that exercise using a treadmill during 6–14 weeks of age had lower body weights than non‐exercised rats. However, fat mass increased during the 4‐week detraining period, and body weight returned to the same level as that of non‐exercised rats (Sertie et al., 2019). By contrast, Otsuka Long‐Evans Tokushima Fatty (OLETF) rats which had increased exercise levels by voluntary wheel running during 4–16 weeks of age did not become overweight after a 4‐week inactivity period without voluntary wheel running (Linden et al., 2013). OLETF rats are recognized as an animal model of obesity that demonstrates hyperphagia owing to a deficit in the cholecystokinin (CCK)‐1 receptor gene (Bi & Moran, 2016). The absence of CCK‐1 receptors in the gastrointestinal tract and brain results in hyperphagia, and consequently, in developing obesity. Although OLETF rats exhibit diabetic complications, such as ocular, renal, and neural lesions, their development is slower than that of other animal models of obesity (Katsuda et al., 2014). The slow progression of diabetic complications in OLETF rats is suitable to simulate the natural history of diabetes in humans with obesity. Regular exercise using voluntary wheel running reduced body weight in obese adult OLETF rats (Moran & Bi, 2006). Similarly, in young OLETF rats, regular exercise by voluntary wheel running decreased food intake and relieved body weight gain with aging (Moran, 2008). However, food intake and body weight increased during the detraining period, induced by locking the running wheel. Thus, obesity and metabolism during the detraining period may depend on the durations of exercise and detraining during the young period. However, few studies have focused on the length of the exercise or detraining periods.

This study aimed to clarify the effects of short‐ and long‐term exercise during the young period on obesity‐related pathological changes in adult obese animals after cessation of exercise. We focused on the histopathological changes at the time point that shows the weight gain and adiposity with cessation of the exercise. Histological changes were observed in the pancreas, WAT, and liver as fibrosis, hypertrophy, and steatosis, respectively. The changes could be different between short‐ and long‐term exercises during the young period, despite a similar level of body weight in the following non‐exercise period. Environmental exposures during early life can permanently impact health and disease in later life, which is known as Barker's hypothesis and the “developmental origins of health and disease” theory (Barker & Osmond, 1986; Shrestha et al., 2020). Understanding the effects of exercise during the young period and cessation of the exercise on obesity in adulthood may be valuable for preventing and treating obesity and its related health problems.

## 2 METHODS

### 2.1 Animals

**FIGURE 1:**
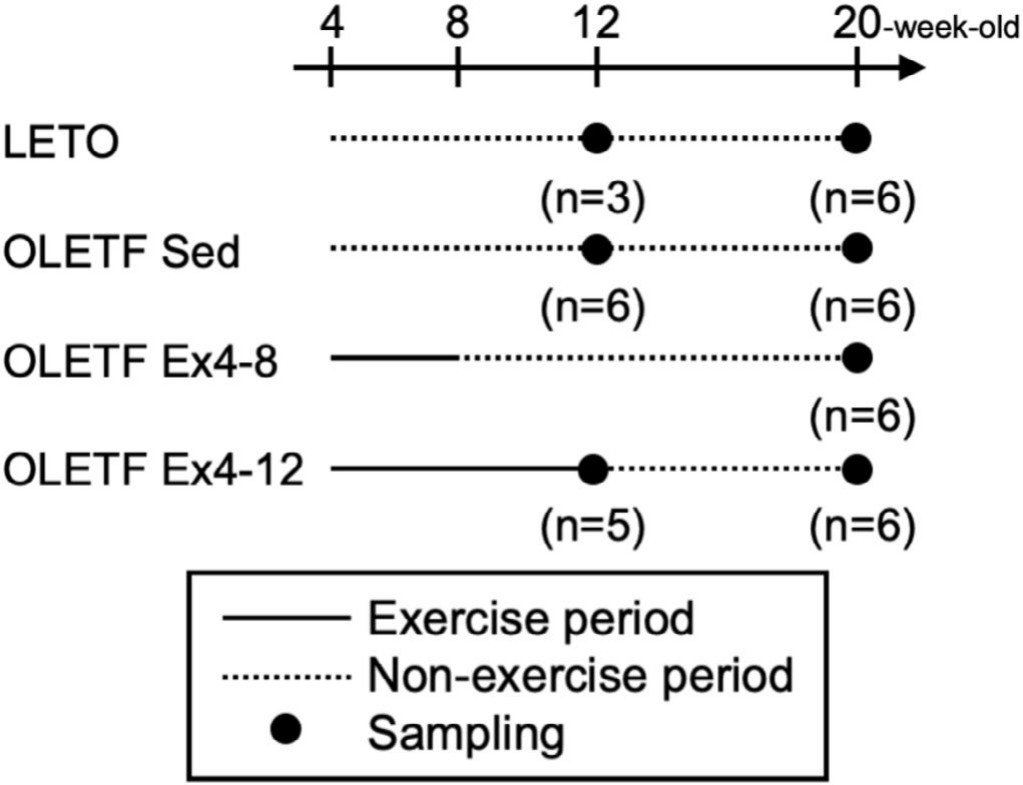
Experimental design. LETO, Long‐Evans Tokushima Otsuka rat; OLETF, Otsuka Long‐Evans Tokushima Fatty rat. LETO (*n* = 9: Tissue samples of 3 and 6 rats were obtained at 12‐ and 20‐week‐old, respectively); OLETF Sed (OLETF non‐exercise sedentary, *n* = 12: Tissue sampling of 6 rats each was performed at 12‐ and 20‐week‐old); OLETF Ex 4–8 (OLETF exercise from 4‐ to 8‐week‐old and detraining from 8‐ to 20‐week‐old, *n* = 6: Tissue samples of 6 rats each was obtained at 20‐week‐old); OLETF Ex 4–12 (OLETF exercise from 4‐ to 12‐week‐old and detraining from 12‐ to 20‐week‐old, *n* = 11: Tissue samples of 5 and 6 rats was obtained at 12‐ and 20‐week‐old, respectively). The solid and dotted lines indicate exercise and non‐exercise periods, respectively. Dark circles indicate the points of tissue sampling.

All rats were purchased from the Japan SLC (Shizuoka, Japan) at 4 weeks old. Previous studies have used approximately 4‐week‐old rats as representative of childhood obesity in humans (Aguilar‐Lozano et al., 2025; Mizuno et al., 2023; Usluel et al., 2023). In addition, 21–60‐day‐old rats were used to simulate the childhood growth stage, and this period represented childhood to adolescence in humans (Mizuno et al., 2023). Therefore, 4‐week‐old male OLETF rats (*n* = 29) and age‐matched male Long‐Evans Tokushima Otsuka (LETO) rats (*n* = 9: tissue sampling of 3 and 6 rats was performed at 12 and 20 weeks of age, respectively) were used as obesity and non‐obesity model animals, respectively. The OLETF rats were divided into the sedentary (OLETF Sed, *n* = 12: tissue sampling of 6 rats each was performed at 12 and 20 weeks of age) and exercise groups by lottery grouping randomization. The rats in the exercise group were further divided into two subgroups according to the exercise period: exercised from 4 to 8 weeks and non‐exercised from 8 to 20 weeks of age (OLETF Ex 4–8, *n* = 6), and exercised from 4 to 12 weeks and non‐exercised from 12 to 20 weeks of age (OLETF Ex 4–12, *n* = 11: tissue sampling of 5 and 6 rats was performed at 12 and 20 weeks of age, respectively) (Figure 1).

Three rats were placed in a cage and housed in a controlled room with a 12‐h light and dark cycle at a constant temperature of 22 ± 2°C. A standard diet (7.9% moisture, 23.1% crude protein, 5.1% crude fat, 5.8% crude ash, 2.8% crude fiber, and 55.3% nitrogen‐free extract) and water were provided ad libitum. Body weights were measured daily at 8 a.m. Daily food intake was recorded from 8 a.m. to 8 a.m. the next morning. OLETF rats rarely express severe weight loss and poor feeding by stress due to sudden environmental changes, such as noise in transit and aggressive behavior with group feeding. If the rats expressed the specific signs, we excluded the rats from the experiment. The sample size was estimated based on our previous studies (Fujita et al., 2019; Goto et al., 2020). Additionally, the minimum sample size per each group of five was calculated to detect significant differences in the body weight at 20 weeks old (*α* = 0.05, power = 0.8).

### 2.2 Exercise protocol

Voluntary wheel running was applied as physical exercise in the present study. All rats in the exercise groups acclimated to a cage with a locked running wheel for 3 days prior to the exercise period. During the exercise period, the rats in the exercise groups were housed individually in a cage with a running wheel for 12 h (20:00–8:00) daily, where they could run voluntarily at any time on the running wheel. Non‐exercised rats were also housed individually in a cage without a running wheel through the dark period. All rats, including non‐exercised rats, were relocated to individual cages through the dark period. Food and water were provided ad libitum in the cages with the running wheels. The ambient temperature was maintained at the same temperature as that of the home cages. The running time, distance, and average and maximum running speeds were automatically recorded using magnetic sensors attached to the running cage and stored in a corresponding digital meter (Figure S1).

Incidentally, almost all male OLETF rats achieved weaning and spermarche by 4 and 12 weeks of age, respectively, which has been ascertained from our past studies (data not shown). In fact, we confirmed the achievements in all rats of the present study. In addition, almost all male OLETF rats exhibited severe hyperglycemia by 20 weeks of age (Kawano et al., 1992). Mizuno et al. used 21–60‐day‐old, 61–100‐day‐old, and 101–140‐day‐old rats to simulate childhood to adolescence, young adulthood, and adulthood in humans (Mizuno et al., 2023). Therefore, when applied to human cases, the exercise protocols, including the exercise period from 4‐ to 12‐week‐old and non‐exercise period from 12‐ to 20‐week‐old, imitate regular exercise in school‐aged children and young adults and discontinuation of exercise in adults, respectively.

### 2.3 Tissue sampling

At the beginning of the light period (i.e., around 8 a.m.), the rats were euthanized at 12 or 20 weeks of age by an overdose of sodium pentobarbital (500 mg/kg body weight). Casual blood glucose levels, not necessarily fasting, were measured in the lateral caudal vein before sacrifice using an ACCU‐CHECK ST meter (Roche, Tokyo, Japan). Blood samples were collected from the inferior vena cava and centrifuged at 3000 *× g* for 10 min to quantify plasma insulin and TG levels. Insulin levels were measured using an enzyme‐linked immunosorbent assay kit (M1101; Morigana, Yokohama, Japan). TG levels were measured using a spectrophotometric assay kit (290–63701; Wako, Osaka, Japan). The epididymal WAT, liver, pancreas, and gastrocnemius muscle were collected. The epididymal fat was selected as representative WAT that showed predominant obesity‐induced adipocyte hypertrophy at a young age. The liver and gastrocnemius muscle were frozen in liquid nitrogen and stored at −80°C. The WAT, liver, and pancreas were fixed with 4% paraformaldehyde in 0.1 M phosphate buffer (pH 7.4).

### 2.4 Analysis of skeletal muscle

Muscle samples were homogenized using a disposable homogenizer (BioMasher II, Nippi, Tokyo, Japan) in five volumes of 10 mM HEPES buffer (pH 7.3) containing 11.5% sucrose, 0.1% Triton‐X100, 1 mM dithiothreitol, and 5% protease inhibitor cocktail (25955‐11, Nacalai Tesque, Kyoto, Japan). After centrifugation at 1500 *× g* for 10 min at 4°C, the supernatants were collected. Citrate synthase (CS) activity was measured by spectrophotometry as described by Srere (1969).

Ten micrometer‐thick serial transverse sections of the muscle belly of the gastrocnemius lateralis were prepared using a cryostat at −20°C. The sections were stained with adenosine triphosphatase (ATPase; pH 4.2) and succinate dehydrogenase (SDH) activities to categorize the muscle fibers as type I, IIA, or IIB. Images stained with ATPase (pH 4.2) or SDH were converted to grayscale images, and the optical density of the muscle fibers was measured. For ATPase (pH 4.2) staining, values <150 and >170 were defined as densely and lightly stained fibers, respectively. For SDH staining, values <170 and >180 were defined as densely stained and lightly stained fibers, respectively. Muscle fibers densely stained for both ATPase (pH 4.2) and SDH were categorized as type I, those stained lightly for ATPase (pH 4.2) and densely for SDH were categorized as type IIA, and those stained lightly for both ATPase (pH 4.2) and SDH were categorized as type IIB. Four random fields were selected, and 200 muscle fibers per rat were measured to determine the muscle fiber type distribution.

### 2.5 Analysis of WAT

Paraffin sections of the WAT were prepared using a microtome and stained with hematoxylin and eosin for histological observation. Two hundred adipocytes were measured from four randomly selected images to create a histogram of adipocyte diameter. Adipocytes with >100 μm and <30 μm in diameter were considered hypertrophic and small, respectively.

The sections were also immunohistochemically stained for CD68 to visualize total macrophages. The sections were blocked with Blocking One Histo (Nakalai Tesque, Kyoto, Japan) and incubated at 4°C overnight with CD68 antibodies (1:100, sc‐59103; Santa Cruz Biotechnology, CA, USA). Subsequently, the sections were exposed to HRP‐conjugated secondary antibody (#8125; Cell Signaling, MA, USA) for 30 min at room temperature and then visualized using DAB (#8059; Cell Signaling). Adipocytes surrounded by CD68‐positive macrophages identified by immunohistochemistry were counted as crown‐like structures (CLS).

### 2.6 Analysis of liver

The hepatic TG content was measured using a spectrophotometric assay kit (290–63701; Wako). Total lipids from the liver samples were extracted according to the method described by Folch et al. (1957).

Total RNA was isolated using the TRIzol reagent (15596–026; Invitrogen, Tokyo, Japan). Reverse transcription was performed using a High‐Capacity cDNA Reverse Transcription Kit (4374966; Applied Biosystems, CA, USA), and the resultant cDNA samples were stored at −20°C until the analysis. The procedures were performed according to the instructions in the attached manual. The expression levels of acetyl‐CoA carboxylase (ACC: Rn00588290_m1), acyl‐CoA oxidase (ACO: Rn01460628_m1), tumor necrosis factor‐α (TNF‐α: Rn01525859_g1), and interleukin‐1β (IL‐1β: Rn00580432_m1) mRNA were quantified using quantitative polymerase chain reaction (qPCR) with TaqMan Gene Expression Assays (Applied Biosystems). The relative expression levels of ACC, ACO, TNF‐α, and IL‐1β were inferred by normalizing the quantity of cDNA template for each gene against the quantity of cDNA for the normalization gene 18S (Rn03928990_g1). The cDNA concentration for each qPCR cycle was plotted to obtain a standard curve, and relative gene expression was calculated as the value at the threshold line. qPCR was performed using PCR Fast Advanced Master Mix (Applied Biosystems) in a 96‐well reaction plate. Each well contained a cDNA template, PCR Fast Advanced Master Mix polymerase kit, and TaqMan Gene Expression Assays with the corresponding primers and probes. All samples and non‐template control reactions were performed in CFX96 (Bio‐Rad Laboratories) under the following conditions: 50°C for 2 min and 95°C for 20 s, followed by 40 cycles at 95°C for 3 s and 60°C for 30 s.

Paraffin sections of the liver were prepared using a microtome and stained with hematoxylin and eosin for histological observation. Hepatic steatosis was defined as the percentage of hepatocytes in a 100‐μm square centered on the central vein with macrovesicular or microvesicular lipid drops.

### 2.7 Analysis of pancreas

Paraffin sections of the pancreas were prepared using a microtome and stained using the immunohistochemistry method targeting insulin, and with picrosirius red. In immunohistochemistry, the sections were incubated at 4°C overnight with insulin antibodies (1:200, sc‐8033; Santa Cruz Biotechnology) to identify B cells of the islets of Langerhans. Subsequently, the sections were exposed to an HRP‐conjugated secondary antibody (Cell Signaling Technology) for 30 min at room temperature and then visualized using DAB (Cell Signaling Technology). Immunohistochemistry‐stained images were converted to grayscale images, and the optical density of the islet region was measured. Areas with values <120 were defined as insulin‐positive regions. Images stained with picrosirius red were also converted to grayscale. The optical density of the islet region was measured, and areas with values <175 were defined as the picrosirius red‐positive regions.

All histological parameters of the skeletal muscle, WAT, liver, and pancreas were quantified using the ImageJ software (NIH, Bethesda, MD, USA). All tissue samples were assigned a number to blind the experimental group allocation.

### 2.8 Statistics

Data are expressed as means ± standard deviations. Significant differences between groups were evaluated using a one‐way analysis of variance with the Tukey post hoc test. Statistical significance was set at *p* < 0.05. All statistical analyses were performed using the SPSS statistical analysis software (IBM SPSS Statistics version 19.0; IBM Japan, Tokyo, Japan).

## 3 RESULTS

### 3.1 Changes in body weight and food intake

**FIGURE 2:**
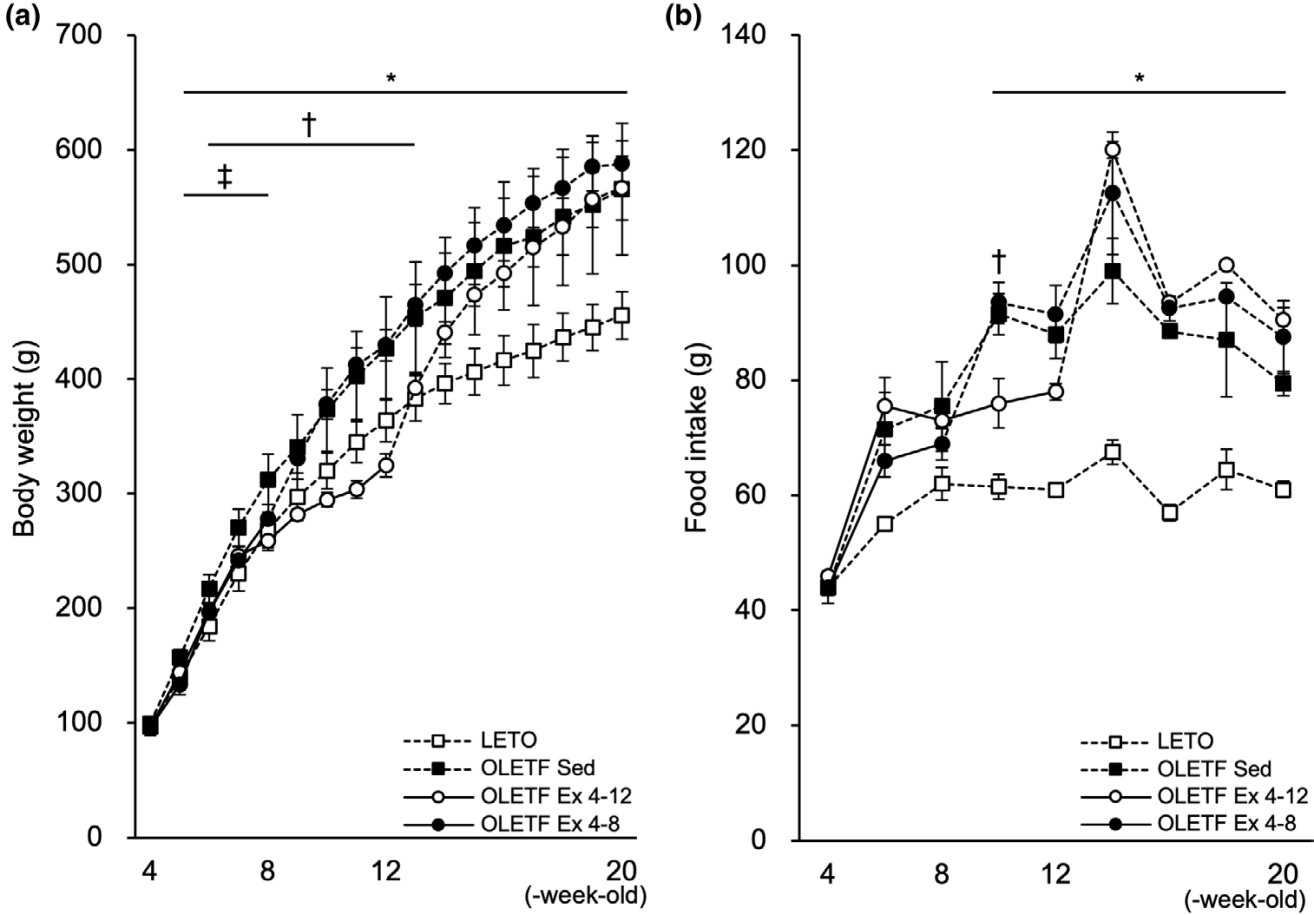
Effects of exercise and detraining during the young period on body weight. The body weights and food intake were significantly lower in the OLETF Ex 4–12 and OLETF Ex 4–8 groups than in the OLETF Sed group throughout the exercise period. However, the values in the OLETF Ex 4–12 and OLETF Ex 4–8 groups increased during detraining period and did not differ from those of the OLETF Sed group at 20‐week‐old. (a) Changes in body weight. (b) Daily food intake (per three rats in a cage). Values represent means ± standard deviations. Solid and dotted lines indicate exercise and non‐exercise periods, respectively. *, †, and ‡ show significant differences between the OLETF Sed group versus LETO rats, the OLETF Sed versus OLETF Ex 4–12 groups, the OLETF Sed versus OLETF Ex 4–8 groups, respectively, *p* < 0.05. The results of the one‐way analysis of variance with the Tukey post hoc test are displayed. *N* = 5–6 per group.

At 4‐week‐old, there were no significant differences in the body weight (Figure 2a) or food intake (Figure 2b) between the groups. After 5‐week‐old, the body weight in the OLETF Sed group was significantly higher than that in the LETO rats (*p* < 0.05). Food intake gradually increased in the OLETF Sed group and was significantly higher than that in the LETO rats after 10 weeks old (*p* < 0.05).

During the exercise period, the food intake of the exercise group was lower than that of the OLETF Sed group; however, it increased rapidly after the exercise period, and they consumed as much food as the OLETF Sed group during the detraining period. The body weights in the OLETF Ex 4–8 group were significantly lower than that in the OLETF Sed group aged 5–8 weeks (i.e., during the exercise period) (*p* < 0.05). However, their body weights increased rapidly after the exercise period and did not differ from those in the OLETF Sed group aged 9‐ to 20 weeks.

The body weights in the OLETF Ex 4–12 group were also significantly lower than that in the OLETF Sed group throughout the exercise period and in the first week of the detraining period (*p* < 0.05). However, their body weights increased rapidly during detraining and did not differ from those of the OLETF Sed group after 14 weeks old. There was no significant difference in the body weight between the OLETF Ex 4–12 and Ex 4–8 groups.

### 3.2 Histological changes in the skeletal muscle

**FIGURE 3:**
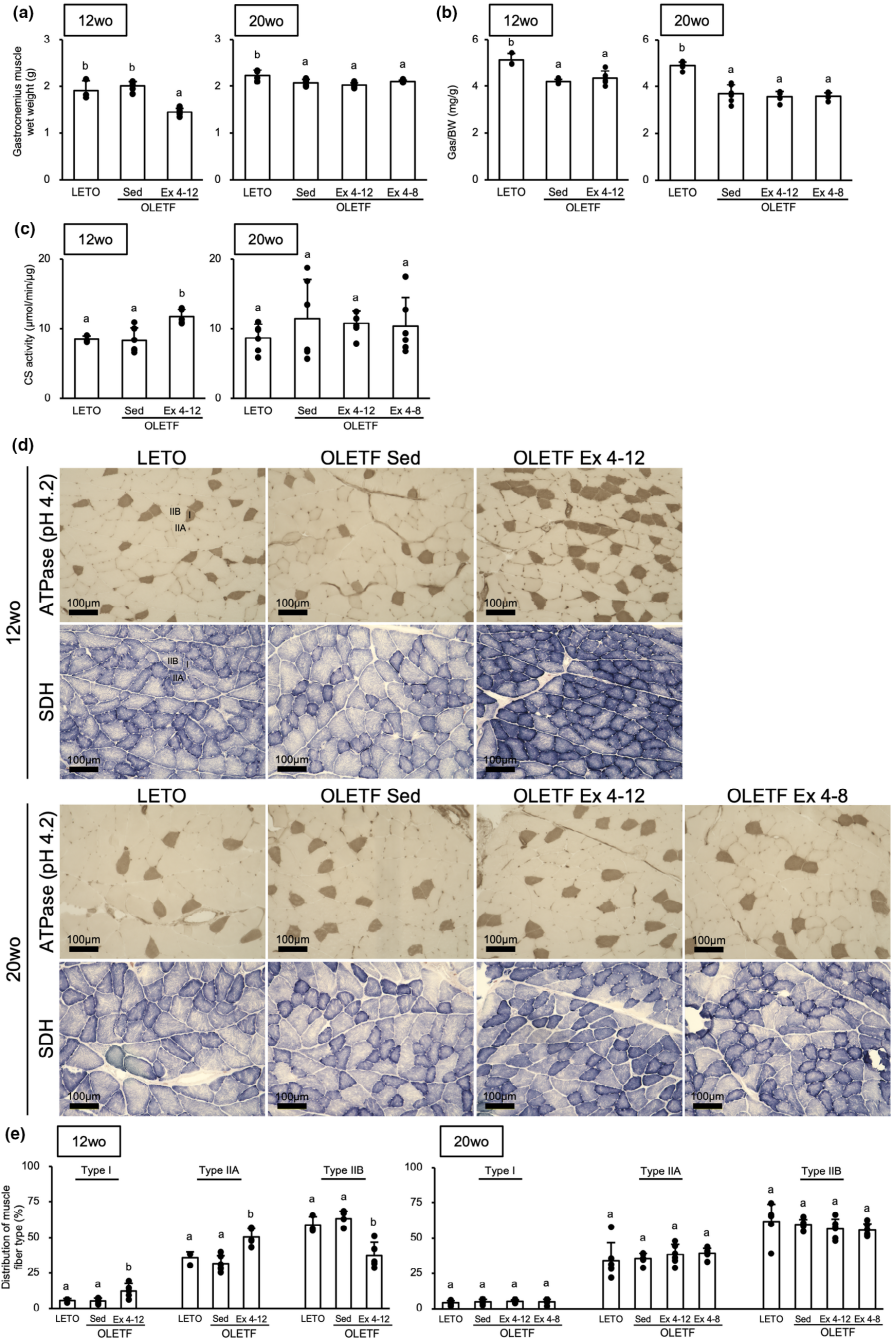
Effects of exercise and detraining during the young period on the skeletal muscle. The CS activity and type I and IIA fiber distribution in the gastrocnemius muscle were significantly higher in the OLETF Ex 4–12 group than in the OLETF Sed group at 12‐week‐old precisely after the exercise period. However, at 20‐week‐old after the detraining period, there was no significant difference in the values across the groups. (a) Gastrocnemius muscle wet weight. (b) Gastrocnemius muscle wet weight relative to body weight. (c) CS activity. (d) Representative sections of the gastrocnemius lateralis. (e) Distribution of each muscle fiber type. ATPase, adenosine triphosphatase; CS, citrate synthase; SDH, succinate dehydrogenase; wo, week‐old. I, IIA, and IIB indicate type I, IIA, and IIB fibers, respectively. Values represent means ± standard deviations. ^a,b^Different superscript letters show significant differences among groups at *p* < 0.05. The results of the one‐way analysis of variance with the Tukey post‐hoc test are displayed. *N* = 3–6 per group.

At 12‐week‐old, the wet weight of the gastrocnemius muscle did not differ between the OLETF Sed group and LETO rats (Figure 3a). However, the muscle weight relative to body weight was significantly lower in the OLETF Sed group than in the LETO rats (*p* < 0.001) (Figure 3b). The wet weight of the gastrocnemius muscle of the OLETF Ex 4–12 group was significantly lower than that of the OLETF Sed group (*p* < 0.001). However, there was no significant difference in the muscle weight relative to body weight between the OLETF Sed and OLETF Ex 4–12 groups. At 20‐week‐old, these values of the OLETF Sed, Ex 4–12, and Ex 4–8 groups were significantly lower than those of the LETO rats (*p* < 0.05), and there were no significant differences among the OLETF Sed, Ex 4–12, and Ex 4–8 groups.

At 12‐week‐old, the CS activity in the gastrocnemius muscle was significantly higher in the OLETF Ex 4–12 group than in the LETO rats and OLETF Sed group (*p* < 0.05) (Figure 3c). However, there were no significant differences in CS activity between the groups at 20‐week‐old.

The gastrocnemius muscle was composed of type I, IIA, and IIB fibers in all the groups (Figure 3d). At 12 weeks old, the type I and type IIA fiber distribution in the OLETF Ex 4–12 group was significantly higher than those in the LETO rats and OLETF Sed group (*p* < 0.05) (Figure 3e). The distribution of type IIB fibers was significantly lower in the OLETF Ex 4–12 group than those in the LETO rats and OLETF Sed group (*p* < 0.01). However, at 20 weeks old, there was no significant difference in the muscle fiber type distribution across the groups.

### 3.3 Changes in metabolism

**FIGURE 4:**
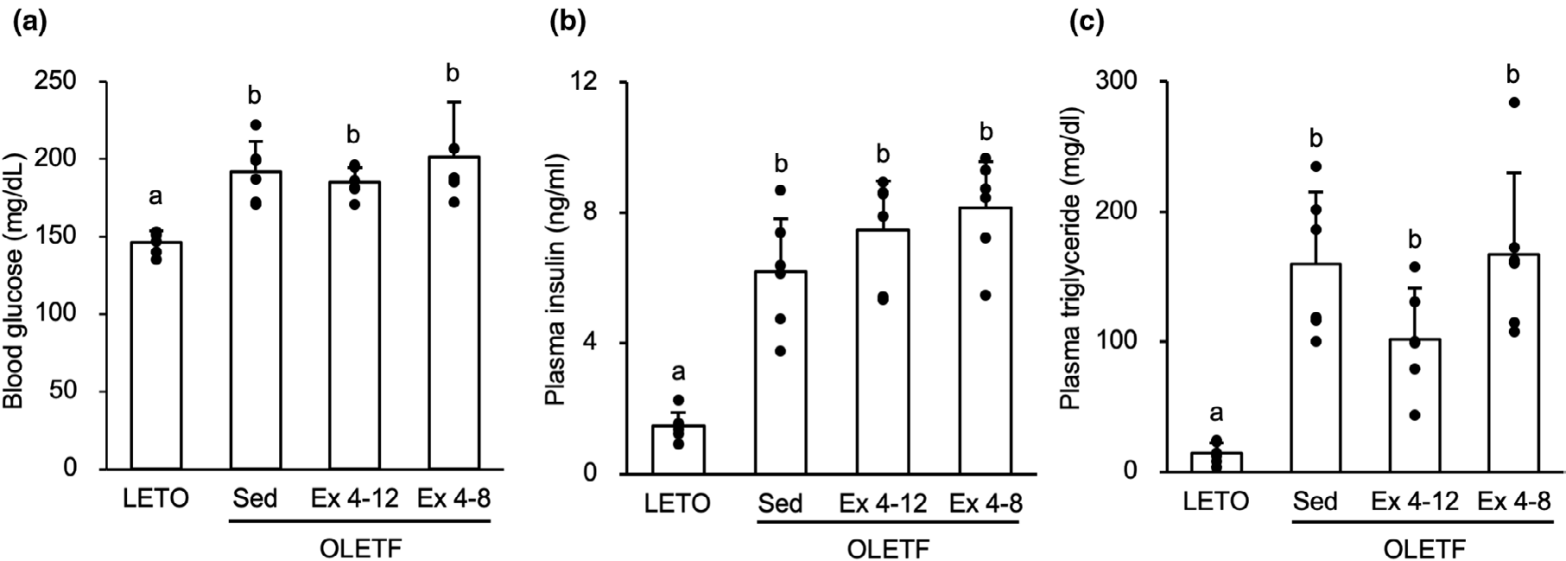
Effects of exercise and detraining during the young period on metabolism. There were no significant differences in blood glucose, plasma insulin, and plasma triglyceride levels among the OLETF Sed, Ex 4–12, and Ex 4–8 groups at 20‐week‐old. (a) Casual blood glucose regardless of when rats last ate. (b) Plasma insulin. (c) Plasma triglyceride. Values are at 20‐week‐old and represent means ± standard deviations. ^a,b^Different superscript letters show significant differences among groups at *p* < 0.05. The results of the one‐way analysis of variance with the Tukey post hoc test are displayed. *N* = 5–6 per group.

At 12‐week‐old, for the casual blood glucose level regardless of when the rats last ate, the value in the OLETF Ex 4–12 group was 138.0 ± 11.3 mg/dL, and similar to that in the LETO rats. In contrast, at 20‐week‐old, for the casual blood glucose levels in the OLETF Sed, OLETF Ex 4–8, and OLETF Ex 4–12 groups were significantly higher than that in the LETO rats (*p* < 0.05) (Figure 4a). There was no significant difference in the casual blood glucose level between the OLETF Ex 4–12 and Ex 4–8 groups.

Plasma insulin (Figure 4b) and TG (Figure 4c) levels were significantly higher in the OLETF Sed group than those in the LETO rats (*p* < 0.05). There was no significant difference among these levels in the OLETF Sed, Ex 4–12, and Ex 4–8 groups.

### 3.4 Histological changes in the WAT

**FIGURE 5:**
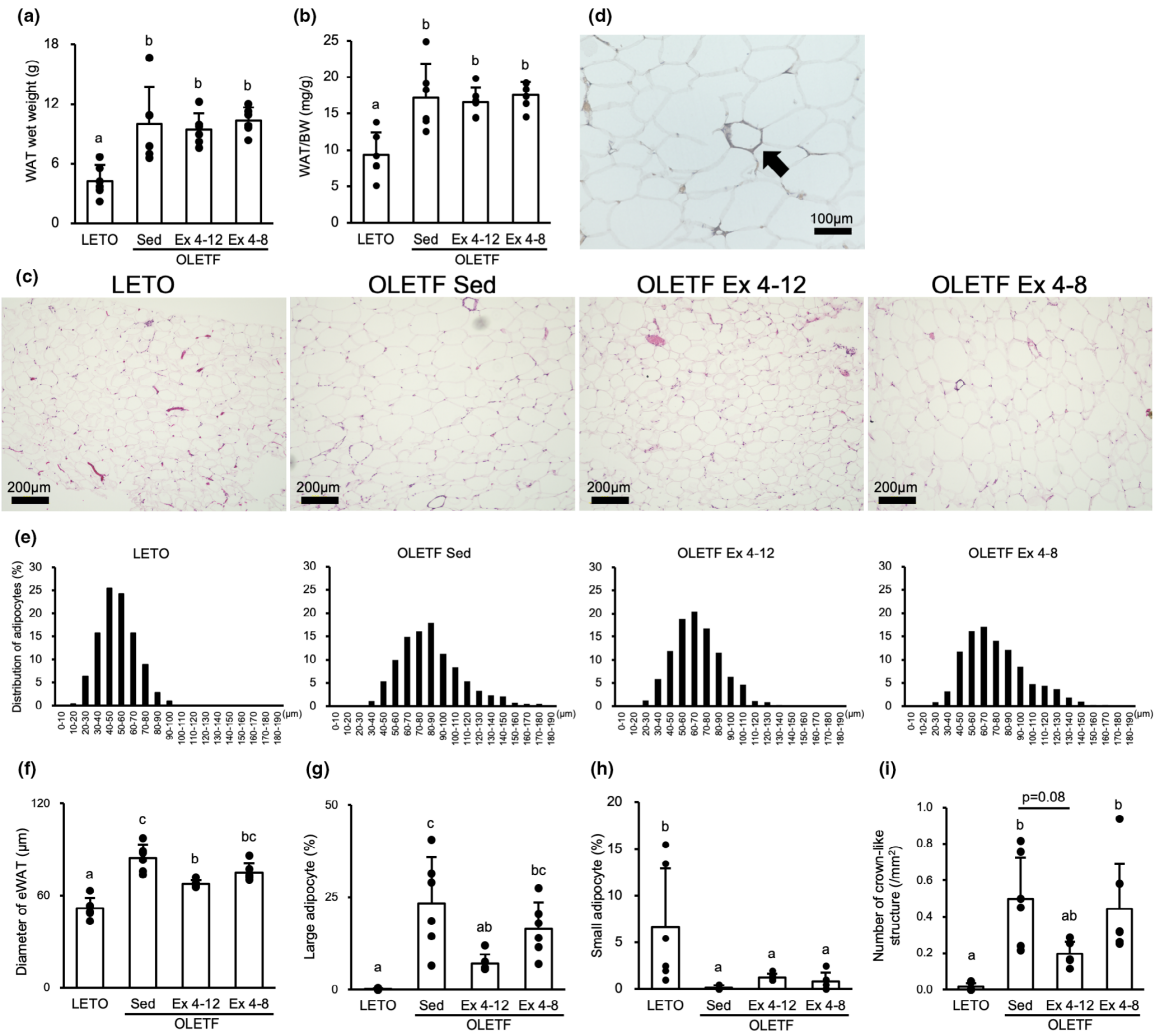
Effects of exercise and detraining during the young period on the epididymal white adipose tissue. There was no significant difference in the wet weight of WAT among the OLETF Sed, Ex 4–12, and Ex 4–8 groups at 20‐week‐old. However, the adipocyte diameter, percentage of enlarged adipocytes, and number of CLS were lower in the OLETF Ex 4–12 group than in the OLETF Sed group. (a) WAT wet weight. (b) WAT wet weight relative to body weight. (c) Representative sections of the WAT stained with hematoxylin and eosin. (d) Representative crown‐like structure images immunostained for CD68. (e) Distribution of adipocyte diameter. (f) Adipocyte diameter of WAT. (g) Percentage of enlarged adipocytes. (h) Percentage of small adipocytes. (i) Number of crown‐like structures. WAT, epididymal white adipose tissue. Values are at 20‐week‐old and represent means ± standard deviations. ^a,b,c^Different superscript letters show significant differences among groups at *p* < 0.05. The results of the one‐way analysis of variance with the Tukey post hoc test are displayed. *N* = 5–6 per group.

At 20‐week‐old, the wet weight of WAT and the ratio to body weight were significantly higher in the OLETF Sed group than those in the LETO rats (*p* < 0.01) (Figure 5a,b). There was no significant difference among the OLETF Sed, Ex 4–12, and Ex 4–8 groups.

Histological appearances and immunohistochemical staining of CD68 visualized CLS were shown in Figure 5c,d, respectively. The histological findings implied adipocyte hypertrophy and chronic inflammation in OLETF rats. Because the findings of more hypertrophied adipocytes and frequent occurrence of CLS in OLETF rats were simply microscopic observations, the adipocyte diameter and number of CLS (i.e., CD68‐positive macrophages) were measured to quantify the observations. The histogram of the adipocyte diameter is presented in Figure 5e. The mean adipocyte diameter (Figure 5f) was significantly higher in the OLETF Sed group (85 ± 26; skewness, 0.81; kurtosis, 0.83) than in the LETO rats (68 ± 20; skewness, 0.54; kurtosis, 0.44) with right‐skewed distribution. However, in the OLETF Ex 4–12 (68 ± 20; skewness, 0.54; kurtosis 0.44) and Ex 4–8 (75 ± 26; skewness, 0.79; kurtosis, 0.38) groups, the distribution of adipocyte diameters shifted to the left compared to that in the OLETF Sed group. The mean adipocyte diameter was lower in the OLETF Ex 4–12 group than that in the OLETF Ex 4–8 group, though the difference was not statistically significant. The percentage of enlarged adipocytes (Figure 5g) was significantly higher in the OLETF Sed group than those in the LETO rats (*p* < 0.001). However, those in the OLETF Ex 4–12 group were significantly lower than those in the OLETF Sed group (*p* < 0.01), and those in the OLETF Ex 4–8 group were not significantly different from those in the OLETF Sed group. The percentage of enlarged adipocytes was lower in the OLETF Ex 4–12 group than that in the OLETF Ex 4–8 group, though the difference was not statistically significant. The percentage of small adipocytes was significantly higher in the LETO rats than those in the OLETF Sed group (*p* < 0.001) (Figure 5h). Meanwhile, there was no significant difference among the OLETF Sed, Ex 4–12, and Ex 4–8 groups.

The number of CLS was similar to the percentage of enlarged adipocytes, which was significantly higher in the OLETF Sed group than that in the LETO group (*p* < 0.01) (Figure 5i). However, the value tended to be lower in the OLETF Ex 4–12 group than that in the OLETF Sed group (*p* = 0.08). The number of CLS in the OLETF Ex 4–8 group was not significantly different from that in the OLETF Sed group. The number of CLS was lower in the OLETF Ex 4–12 group than that in the OLETF Ex 4–8 group, though the difference was not statistically significant.

### 3.5 Histological changes in the liver

**FIGURE 6:**
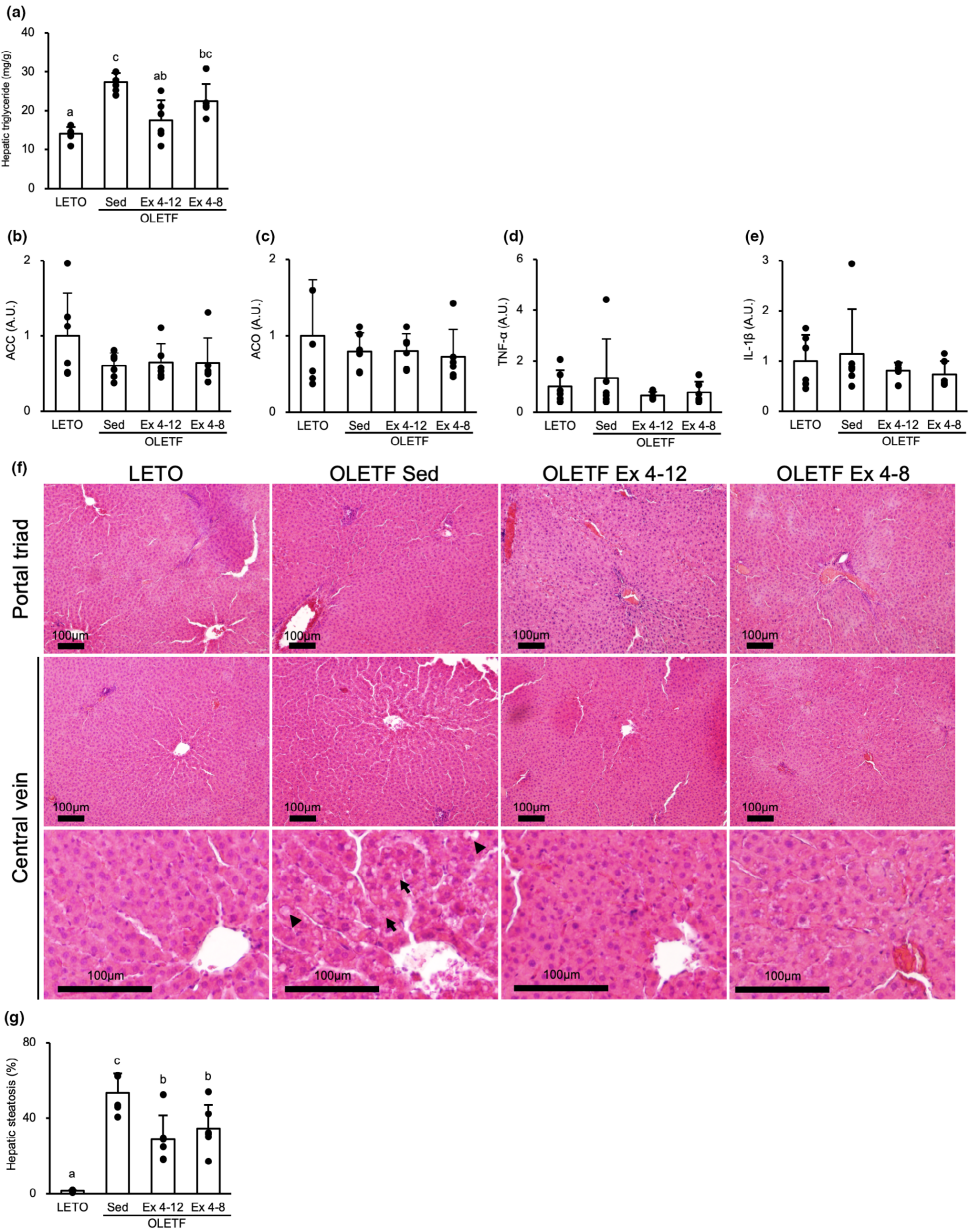
Effects of exercise and detraining during young period on the liver. At 20‐week‐old, the hepatic TG content and percentage of hepatic steatosis were significantly lower in the OLETF Ex 4–12 group than in the OLETF Sed group. (a) Hepatic triglyceride contents. (b–e) mRNA expression level of ACC, ACO, TNF‐α, and IL‐1β, respectively. (f) Representative sections of the liver stained with hematoxylin and eosin around the triad (upper) and central vein in low (middle) and high magnifications (lower). (g) Percentage of hepatic steatosis. ACC, acetyl‐CoA carboxylase; ACO, acyl‐CoA oxidase; IL‐1β, interleukin‐1β; TNF‐α, tumor necrosis factor‐α. A black arrowhead and a black arrow indicate macrovesicular and microvesicular steatosis, respectively. Values are at 20‐week‐old and represent means ± standard deviations. ^a,b,c^Different superscript letters show significant differences among groups at *p* < 0.05. The results of the one‐way analysis of variance with the Tukey post hoc test are displayed. *N* = 5–6 per group.

The hepatic TG content extracted from the liver homogenate was significantly higher in the OLETF Sed group than that in the LETO rats (*p* < 0.001) (Figure 6a). However, the value was significantly lower in the OLETF Ex 4–12 group than that in the OLETF Sed group (*p* < 0.01). The value in the OLETF Ex 4–8 group was not significantly different from that in the OLETF Sed group. The hepatic TG content was lower in the OLETF Ex 4–12 group than that in the OLETF Ex 4–8 group, though the difference was not statistically significant.

Gene expression levels of ACC, ACO, TNF‐α, and IL‐1β were revealed by qPCR analysis. There were no significant differences in lipid metabolism markers, such as ACC (Figure 6b) and ACO (Figure 6c), and inflammation markers, such as TNF‐α (Figure 6d) and IL‐1β (Figure 6e), between the groups.

Histologically, macrovesicular and microvesicular steatosis was observed only around the central vein and not around the portal triad (Figure 6f). The percentage of hepatic steatosis around the central vein was significantly higher in the OLETF Sed group than that in the LETO rats (*p* < 0.001) (Figure 6g). However, the percentages in the OLETF Ex 4–8 and Ex 4–12 groups were significantly lower than that in the OLETF Sed group (*p* < 0.01). There was no significant difference in the percentage between the OLETF Ex 4–12 and Ex 4–8 groups.

### 3.6 Histological changes in the pancreas

**FIGURE 7:**
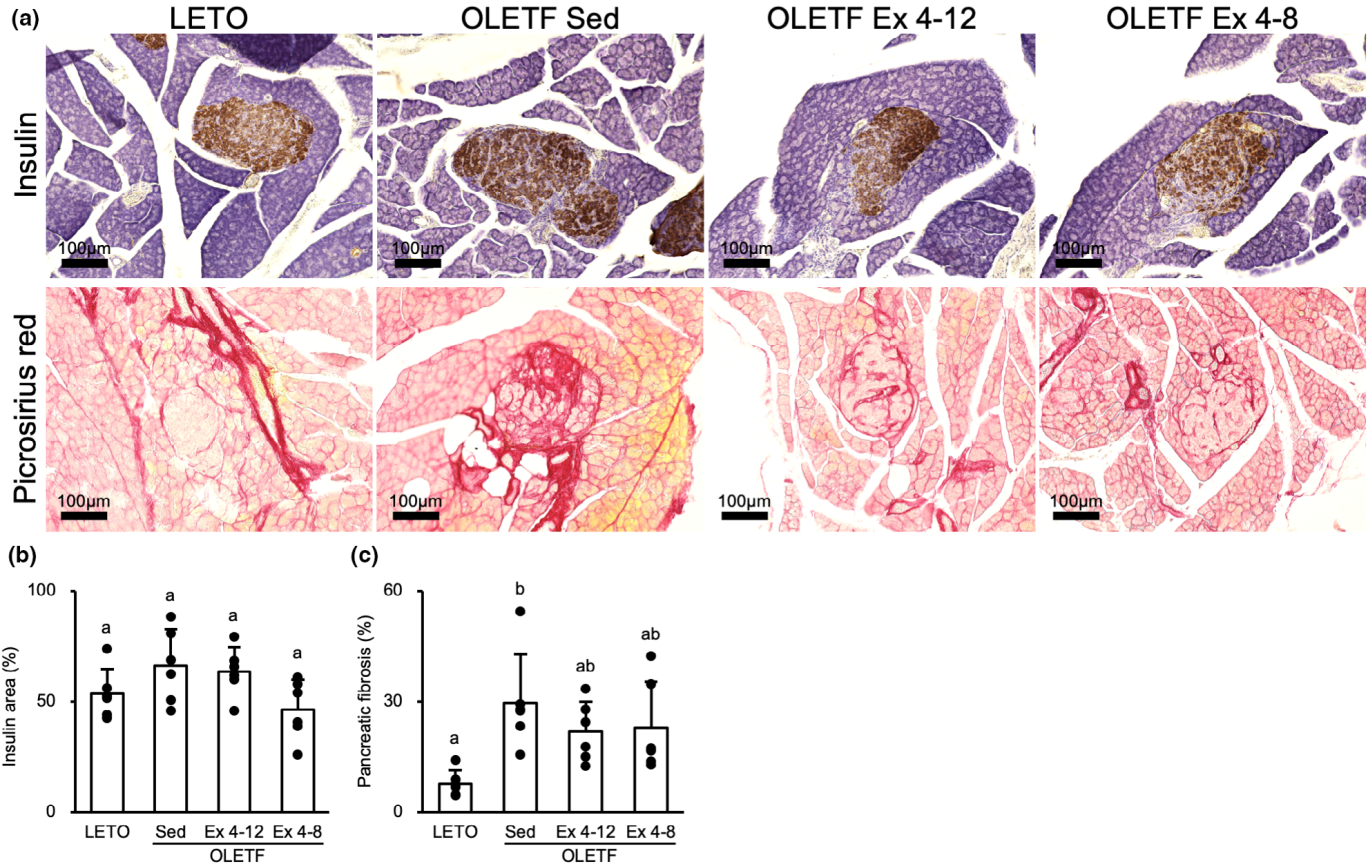
Effects of exercise and detraining during the young period on the pancreas. Although the picrosirius red‐positive area that indicates pancreatic fibrosis was lower in the OLETF Ex 4–8 and Ex 4–12 groups than in the OLETF Sed group, there was no significant difference among the OLETF Sed, Ex 4–12, and Ex 4–8 groups at 20‐week‐old. (a) Representative sections of the pancreas immunostained for insulin (upper) and stained with picrosirius red (lower). (b) Percentage of insulin‐positive area in the islets of Langerhans. (c) Percentage of fibrosis in the islets of Langerhans. Values are at 20‐week‐old and represent means ± standard deviations. ^a,b^Different superscript letters show significant differences among groups at *p* < 0.05. The results of the one‐way analysis of variance with the Tukey post hoc test are displayed. *N* = 5–6 per group.

Visualization of insulin secretion using immunohistochemistry showed that the islets of Langerhans in each group were similarly stained (Figure 7a). The insulin‐positive area was not significantly different between the groups (Figure 7b). In contrast, the visualization of fibrosis by picrosirius red staining showed that the islets in the OLETF Sed, Ex 4–12, and Ex 4–8 groups stained denser than those in LETO rats. Particularly, the islets of the OLETF Sed group were more densely stained. The picrosirius red‐positive area was measured to quantify a denser stained islet based on microscopic observation. The picrosirius red‐positive area of the islets was significantly larger in the OLETF Sed group than that in the LETO rats (*p* < 0.01) (Figure 7c). Although the mean values of the OLETF Ex 4–8 and Ex 4–12 groups were lower than that of the OLETF Sed group, there were no significant differences among the OLETF Sed, Ex 4–12, and Ex 4–8 groups.

## 4 DISCUSSION

Voluntary wheel running during sexual maturation prevented excessive weight gain in male OLETF rats. However, after the detraining period without voluntary wheel running, these benefits diminished with age, and by adulthood (20 weeks of age), all OLETF rats showed similar levels of weight gain regardless of how long they had exercised when young. Despite this, exercised OLETF rats still showed some protection against adipose tissue chronic inflammation, fatty liver, and pancreatic damage compared to non‐exercised OLETF rats, even after detraining. The positive effects on adipose tissue, such as inhibitions of adipocyte hypertrophy and CLS, were weaker in OLETF rats that exercised for shorter periods and had longer detraining periods. This suggests that while body weight eventually became similar, the length and consistency of early‐life exercise influenced the long‐term health of organs involved in energy storage, highlighting that exercise during youth can protect organ health beyond just controlling weight.

### 4.1 Muscle fiber type transitions with exercise during the young period are worn off with detraining

The skeletal muscles of OLETF rats did not exhibit hypertrophy during voluntary wheel running. At 12 weeks old, although the muscle weight did not differ between the OLETF Sed group and LETO rats, the muscle weight relative to body weight was significantly lower in the OLETF Sed group than that in the LETO rats. This means the muscle mass in non‐exercised OLETF rats was not commensurate with the body weight. Meanwhile, although the muscle weight was significantly lower in the OLETF Ex 4–12 group than in the OLETF Sed group, there was no significant difference in the muscle weight relative to body weight between the OLETF Sed and OLETF Ex 4–12 groups. The muscle mass in exercised OLETF rats remained low, which could be attributable to low‐intensity exercise using voluntary wheel running. This voluntary wheel running caused a transition of the muscle fiber type from glycolytic to oxidative and an increase in CS activity in the skeletal muscle. Type I and IIA fibers are rich in mitochondria and have a high oxidative metabolic capacity that facilitates efficient energy consumption (Maltin, 2008). The efficiency of energy metabolism in the skeletal muscles improved during the exercise period with voluntary wheel running, which could prevent excessive weight gain during this period. However, the beneficial effects of exercise on skeletal muscles were attenuated during the detraining period without voluntary wheel running. Therefore, the rats may not have been able to maintain a non‐obese body.

### 4.2 Adipocyte hypertrophy and CLS could be suppressed with long‐term exercise during the young period compared to that with short‐term exercise

The effects of exercise and detraining during the young period on WAT depended on exercise duration. At 20 weeks old in this study, long‐term exercise using voluntary wheel running inhibited adipocyte hypertrophy and chronic inflammation and led to a decrease in the number of hypertrophied adipocytes in the WAT. Short‐term exercise slightly inhibited adipocyte hypertrophy, but not chronic inflammation. Despite the suppression of adipocyte hypertrophy both in the exercise groups, the WAT weights in the exercise groups were not different from that in the sedentary group. WAT growth involves adipocyte proliferation and hypertrophy, with proliferation predominating until adulthood. However, excessive energy intake during childhood leads to a predominance of adipocyte hypertrophy, which increases the risk of metabolic diseases (Corvera et al., 2022; Pouteau et al., 2008). In the OLETF Ex 4–12 group, relatively long‐term exercise could have inhibited adipocyte hypertrophy and caused lipid accumulation with normal adipocyte proliferation during the early growth period. Thus, small adipocytes with hyperplasia can adapt to excess energy intake during the detraining period. In contrast, in the OLETF Ex 4–8 group, insufficient exercise in the early growth period could mainly adapt to adipocyte hypertrophy for excess energy intake during the non‐exercise period. Hence, hypertrophied adipocytes without normal hyperplasia in the early growth period could not adapt to excess energy intake during the detraining period, and redundant energy could not be stored only in the adipose tissue. In addition, CLS were observed with the appearance of hypertrophied adipocytes, mainly in the OLETF Ex 4–8 group. CLS is a histological element of chronic inflammation in the WAT, and its appearance is associated with adipocyte hypertrophy (Murano et al., 2008). Hypertrophied adipocytes with energy storage show necrosis‐like abnormalities when they reach their size limit (Strissel et al., 2007). Subsequently, macrophages migrate around necrotic adipocytes for phagocytosis and their activation state shifts from the M2‐polarized state to the M1 proinflammatory state, leading to the secretion of proinflammatory cytokines (Lumeng et al., 2007; Weisberg et al., 2003). Consequently, chronic inflammation can occur locally and systemically. In the OLETF Ex 4–12 group, longer exercise and shorter detraining periods reduced the number of enlarged adipocytes. Sufficient storage for lipid accumulation may have led to a reduction in CLS appearance. Therefore, continuing exercise for a longer period at a young age is necessary to reduce chronic inflammation in the WAT.

### 4.3 Increase in hepatic TG content could be slightly reduced with long‐term exercise during the young period compared to that with short‐term exercise

At 20 weeks old in the detraining period, although the hepatic TG level was considerably reduced with long‐term exercise using voluntary wheel running compared to that with short‐term exercise, almost the same level of liver steatosis was observed in both the long‐term and short‐term exercise groups. Obesity‐related histopathological changes in the liver can be divided into four stages as follows: (1) increase of hepatic TG content, (2) appearance of hepatic steatosis, (3) progression of steatosis, and (4) decrease in steatosis (Sheldon et al., 2014; Song et al., 2013). In the present study, all rats in the OLETF Sed, OLETF Ex 4–8, and OLETF 4–12 groups are considered to have reached the second stage. As lipid metabolism‐related abnormalities and inflammation appear after the third stage, there were no differences in inflammatory markers in the liver between the groups. The difference in the exercise period at a young age could affect only the TG content and not the histology. Nevertheless, reduced hepatic TG content was observed with long‐term rather than short‐term exercise during the young period. Thus, long‐term exercise during the young period could protect future liver health.

### 4.4 Pancreatic fibrosis could be reduced with both long‐term and short‐term exercise during the young period

Although exercise during the young period using voluntary wheel running inhibited pancreatic fibrosis in adulthood, no significant difference was observed between the long‐ and short‐term exercise groups. Obesity‐ and diabetes‐related histopathological changes in the pancreas are classified into three stages as follows: (1) hyperinsulinemia with hypertrophy of the islets of Langerhans (Iwase et al., 2002; Shiba et al., 1998), (2) hyperglycemia with fibrosis of the islets of Langerhans (Goto et al., 2020; Hong et al., 2002; Yoshikawa et al., 2002), and (3) hypoinsulinemia with atrophy of the islets of Langerhans (Kawano et al., 1992). In the present study, all rats in the OLETF Sed, OLETF Ex 4–8, and OLETF 4–12 groups are considered to belong to the early phase of the second stage. In our previous studies (Fujita et al., 2019; Goto et al., 2020), mild hyperglycemia has been appeared in OLETF rats between 4‐ and 8‐week‐old. In addition to hyperglycemia, hypoinsulinemia has been appeared after 20 weeks old. Therefore, generally, the second stage accounts for a long phase at a young age. Although the duration of obesity was shortened by the exercise period, the influences could not be detected owing to bias from having a long duration of the second stage. Indicators including blood glucose and insulin levels and pancreatic histology alone could be insufficient to clarify the differences between the short‐ and long‐term exercise groups.

### 4.5 Differences in food intake between exercise and detraining periods could influence weight gain after exercise cessation

In the present study, voluntary wheel running during the young period prevented excessive weight gain, whereas the body weight could not be maintained through the detraining period regardless of the length of the exercise periods. By contrast, several studies have reported the efficacy of exercise during the young period on obesity after the detraining period. Shima et al. have reported that in OLETF rats, voluntary wheel running during the young period prevented obesity and obesity‐related histopathological changes in the pancreas in adulthood (Shima et al., 1993). In the study using OLETF rats by Linden et al., decreased body weight, visceral fat mass, and hepatic TG content are observed upon voluntary wheel running during the young period, which still show lower levels in adulthood (Linden et al., 2013). Shindo et al. have reported that in OLETF rats, voluntary wheel running from 4‐ to 20‐week‐old prevented excessive body weight gain, which was maintained in adulthood (Shindo et al., 2014). The exercise period in the abovementioned studies is longer than that in the present study (i.e., voluntary wheel running from 4‐ to 12‐week‐old in the present study; from 4‐ to 16‐week‐old in the study by Linden et al.; from 4‐ to 20‐week‐old in the study by Shindo et al.). Additionally, there were differences in food intake among the studies. The food intake during the exercise period was decreased in the present study, and then hyperphagia and excessive body weight gain occurred during the adulthood detraining period. Meanwhile, the food intake in OLETF rats during the exercise period was not decreased in the studies by Linden et al. and Shindo et al., and the low body weight was maintained until adulthood. In the study by Shindo et al., their experimental design contained the pair‐feeding animals that were restricted in the amount of food intake to match the body weight with those of exercised rats. Although the lower body weight in exercised OLETF rats was maintained in the adulthood detraining period, the food intake increased after cessation of pair‐feeding, and body weight gradually increased through adulthood in the pair‐fed OLETF rats. In the study by Chao et al., decreased food intake was noted in OLETF rats exercised by voluntary wheel running during the young period, whereas the food intake and body weight increased through the detraining period (Chao et al., 2011). Cornejo et al. have also reported that hyperphagia induced excessive body weight gain after cessation of pair‐feeding, which caused obesity and glucose intolerance (Cornejo et al., 2020). Thus, excessive body weight gain in the adulthood detraining period could be related to decreased food intake during the exercise period. The effects of regular exercise during the young period in the present study could depend on calorie restriction with decreased food intake, and the detraining in adulthood with hyperphagia could not maintain low body weight resulting from regular exercise during the young period. Therefore, considering these results in human children, the amount of food intake should be observed carefully during the childhood exercise period. In order to maintain a low body weight throughout adulthood, preventing hyperphagia with detraining is important.

## 5 LIMITATIONS

The present study cleared that exercise during the young period attenuated adipocyte hypertrophy, chronic WAT inflammation, and hepatic TG levels in adulthood depending on the duration of exercise, even though there are no differences in body weight between the long‐ and short‐term exercises. Nevertheless, this study had some limitations. The targeted outcome in the present study was only in the early maturation period. This experimental design could contribute that the differences between the long‐ and short‐term exercises during the young period are found only in chronic inflammation of the white adipose tissue, unnoticeable in the liver and pancreas as downstream tissue pathology of chronic inflammation. As the effects of exercise and detraining during the young period were identified only at 20 weeks old, the adaptation process during detraining was not identified. Determining whether the results are attributable to the exercise duration (4 weeks in the OLETF Ex 4–8 group vs. 8 weeks in the OLETF Ex 4–12 group) or detraining duration (12 weeks in the OLETF Ex 4–8 group vs. 8 weeks in the OLETF Ex 4–12 group) was difficult. In other words, a shorter detraining duration may have resulted in more exercise benefits. Future studies should include adequate controls to justify the model and time points. Additionally, the targeted outcome in the present study was observed only at 12‐ or 20‐week‐old, not including at 4‐ or 8‐week‐old. We did not collect the samples in the OLETF Ex 4–8 group at 12 weeks old. No data at 12 weeks old were available, except those on the skeletal muscle, before the exercise period, in the middle of the exercise period, and in the early detraining period. This experimental design could make it difficult to clarify baseline data at 4 weeks old and longitudinal data for discontinuation of exercise at 8 weeks old. Furthermore, only male OLETF rats were analyzed, and the sample size for each group was small. The experimental group in the present study included group‐housed rats. This makes it difficult to clarify the potential sex‐ and strain‐based differences and arises uncertainty regarding how much each rat ate in the group house (daily food intake per each rat may be problematic). Besides food intake, voluntary wheel running was applied as exercise in the present study. Therefore, the exercise protocol, such as intensity and frequency, was not systematically controlled. Further studies are required to clear the limitations.

## 6 CONCLUSION

Shortening the duration of obesity with long‐term exercise at a young age inhibited adipocyte hypertrophy, chronic inflammation, and hepatic fat accumulation in adulthood. This study highlights the importance of long‐term exercise during the young period to prevent not only abnormal metabolic profiles but also obesity‐related pathological changes in adulthood.

## AUTHOR CONTRIBUTIONS

K.S. was involved in formal analysis, investigation, and writing of the original draft. S.U. was involved in resources, supervision, writing, review, and editing. N.F. was involved in conceptualization, data curation, methodology, project administration, resources, supervision, writing—original draft, writing—review, and editing. All the authors have read and approved the final version of the manuscript.

## FUNDING INFORMATION

This study was supported by a Grant‐in‐Aid for Scientific Research from the Japanese Ministry of Education, Culture, Sports, and Technology (22K11393).

## CONFLICT OF INTEREST STATEMENT

There are no conflicts of interest to declare.

## ETHICS STATEMENT

This study was approved by the Institutional Animal Care and Use Committee of Hiroshima University (A19‐163) and was conducted according to the Hiroshima University Regulations for Animal Experimentation. All animal experiments were performed in compliance with the ARRIVE guidelines.

## Supporting information


Figure S1.

Figure S2.

## Data Availability

All data generated or analyzed during this study are included in the paper. Data will be made available upon reasonable request to the corresponding author.
