# Supplementary material for: Exercise duration and detraining influence not only body weight but also histopathological changes in the white adipose tissue of young male OLETF rats as an obesity model
Source: Physiol Rep. 2025 Jul 26;13(14):e70487. doi: 10.14814/phy2.70487 (PMC12296700; doi:10.14814/phy2.70487)
Supplement: Supplementary file 1 — Figure S1. [file PHY2-13-e70487-s001.zip › Supplementary Figure 1.docx]

**Supplementary Figure 1. Exercise parameters**

A: Total running time per day. B: Total running distance per day. C: Average running velocity in a day. D: Maximum running velocity in a day. Values represent means ± standard deviations. The lines with white and dark circles indicate the OLETF Ex 4-12 and OLETF Ex 4-8 groups, respectively.
