# Supplementary figures and images for: Exercise duration and detraining influence not only body weight but also histopathological changes in the white adipose tissue of young male OLETF rats as an obesity model
Source: Physiol Rep. 2025 Jul 26;13(14):e70487. doi: 10.14814/phy2.70487 (PMC12296700; doi:10.14814/phy2.70487)

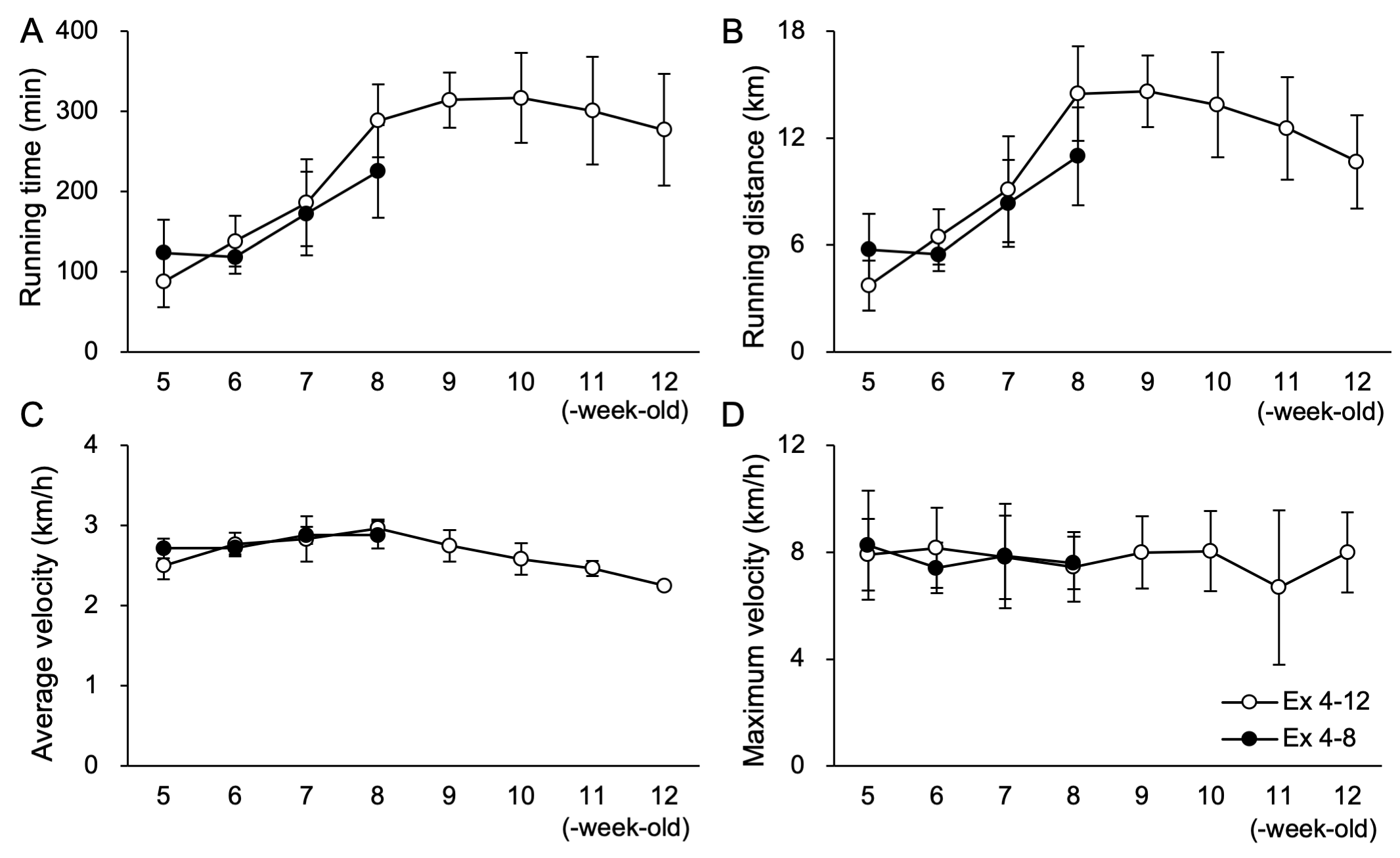

Supplement: Supplementary file 1 — Figure S1. [file PHY2-13-e70487-s001.zip › PHYSREP-2025-01-011-T-f08-z-.tif]
