# Supplementary material for: Exercise duration and detraining influence not only body weight but also histopathological changes in the white adipose tissue of young male OLETF rats as an obesity model
Source: Physiol Rep. 2025 Jul 26;13(14):e70487. doi: 10.14814/phy2.70487 (PMC12296700; doi:10.14814/phy2.70487)
Supplement: Supplementary file 2 — Figure S2. [file PHY2-13-e70487-s002.zip › Supplementary Figure 2.docx]

**Supplementary Figure 2. Metabolic efficiency**

The ratio in the OLETF Ex 4-12 group gradually decreased along with the decrease in the body weight gain during the exercise period. The ratio increased rapidly after exercise cessation along with the increase in both the weight gain and the food intake. However, these values gradually decreased during the detraining period and reached to the level in the OLETF Sed group. The ratio in the OLETF Ex 4-8 group was the same level as that in the OLETF Sed group during the exercise period. Although the ratio transiently continued after exercise cessation, the value gradually decreased during the detraining period. A: The ratio of body weight gain to food intake. B: Body weight gain. C: Food intake. Values represent means. The solid and dotted lines indicate exercise and non-exercise periods, respectively.
